# A case of bowel perforation due to traumatic hernia at a pelvic fracture site: a case report and review of the literature

**DOI:** 10.1186/s12893-017-0278-y

**Published:** 2017-07-12

**Authors:** Ryota Tanaka, Hisashi Nagahara, Kiyoshi Maeda, Hiroshi Ohtani, Masatsune Shibutani, Tatsuro Tamura, Tetsuro Ikeya, Kenji Sugano, Yasuhito Iseki, Katsunobu Sakurai, Sadaaki Yamazoe, Kenjiro Kimura, Takahiro Toyokawa, Ryosuke Amano, Naoshi Kubo, Hiroaki Tanaka, Kazuya Muguruma, Kosei Hirakawa, Masaichi Ohira

**Affiliations:** Department of Surgical Oncology, Osaka City University Graduate School of Medicine, 1-4-3 Asahi-machi, Abeno-ku, Osaka, 545-8585 Japan

**Keywords:** Bowel perforation, Traumatic hernia, Pelvic fracture, Emergency operation

## Abstract

**Background:**

Common complications of pelvic fractures include visceral injury, large-volume hemorrhage, genitourinary injury, rectal injury, and pulmonary embolism. On the other hand, traumatic hernia is a rare complication, especially in association with pelvic fractures. We report a case of bowel perforation due to traumatic hernia at a pelvic fracture site.

**Case presentation:**

A 65-year-old female was presented at our hospital for further examination and treatment of ileus. She was diagnosed with bowel perforation due to traumatic hernia at a pelvic fracture site, and an emergency operation was thus immediately performed. We performed segmental jejunum resection and constructed jejunostomy, and the iliac bone fracture was fixed with four pins. In the postoperative course, she received antibiotics and vasopressors for septic shock. However, there was no need for either a ventilator, dialysis or admission to the ICU. At seven days after the operation, a residual abscess was detected in the pouch of Douglas. We performed percutaneous drainage (Clavien-Dindo IIIa) and jejunostomy closedown 35 days after the first operation. The postoperative course was without complication, but she received rehabilitation until she was able to walk unaided. She was discharged 64 days after the first operation.

**Conclusion:**

The occurrence of traumatic hernia is rare, especially in association with pelvic fractures. Although its rarity, traumatic hernia follows a severe course. Thus, proper diagnosis and effective treatment are necessary. Surgeons treating patients with pelvic injuries should consider the possibility of any complications and perform a work-up examination in order to achieve an accurate diagnosis at an earlier time point.

## Background

Pelvic fractures are often caused by high-energy injuries such as those suffered in traffic accidents and falls, and account for only 5%–8% of all fractures. Common complications of pelvic fractures include visceral injury, hemorrhage, genitourinary injury, rectal injury, and pulmonary embolism. Traumatic hernia is rare, especially in association with pelvic fractures. Although the rarity of such cases, it demonstrates a severe course. Therefore, it requires both a proper diagnosis and treatment.

## Case presentation

**Fig. 1:**
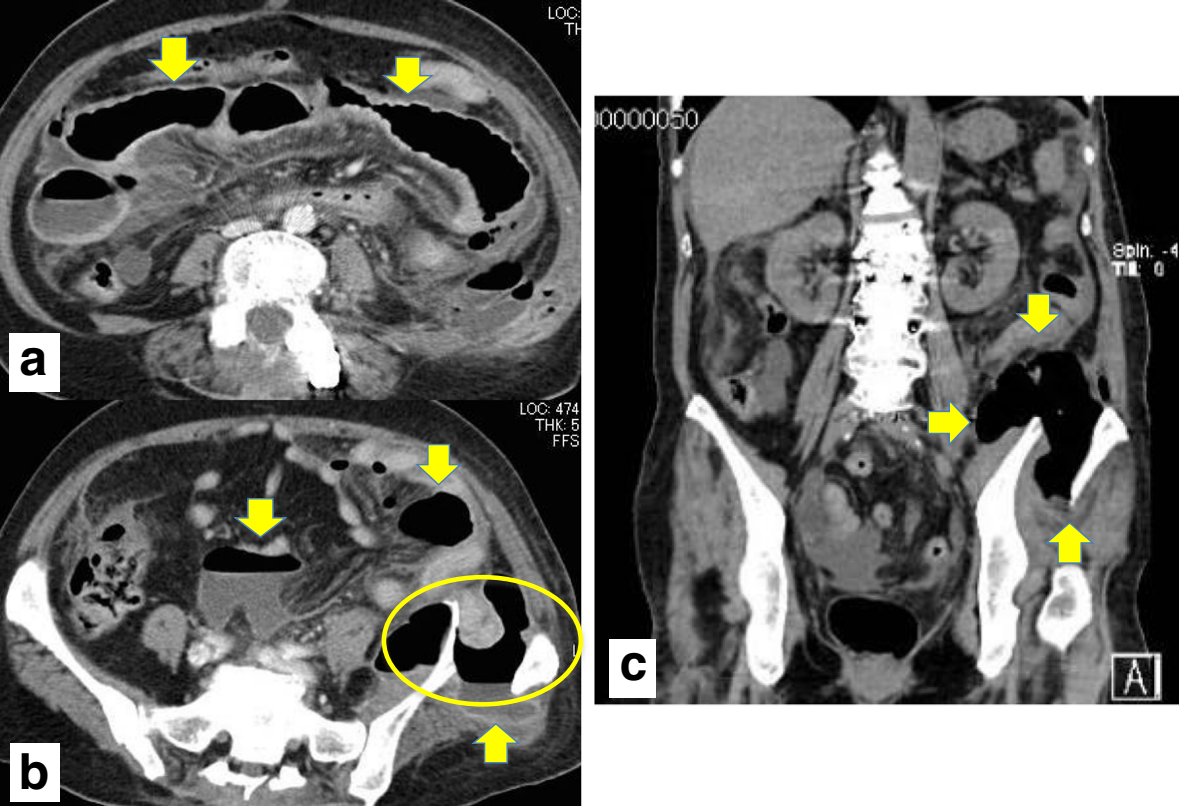
Abdominal contrast-enhanced computerized tomography revealed a left iliac fracture, free air, and herniation at the fracture site. **a** (axial image): Small intestine ileus was shown (arrow). **b** (axial image): Extraintestinal free air and fluid collection were noted (*arrow*). The left iliac wing was fractured, and the small intestine was herniated at the fracture site (circle). **c** (coronal image): Left iliac fracture and free air can be seen (*arrow*)

**Fig. 2:**
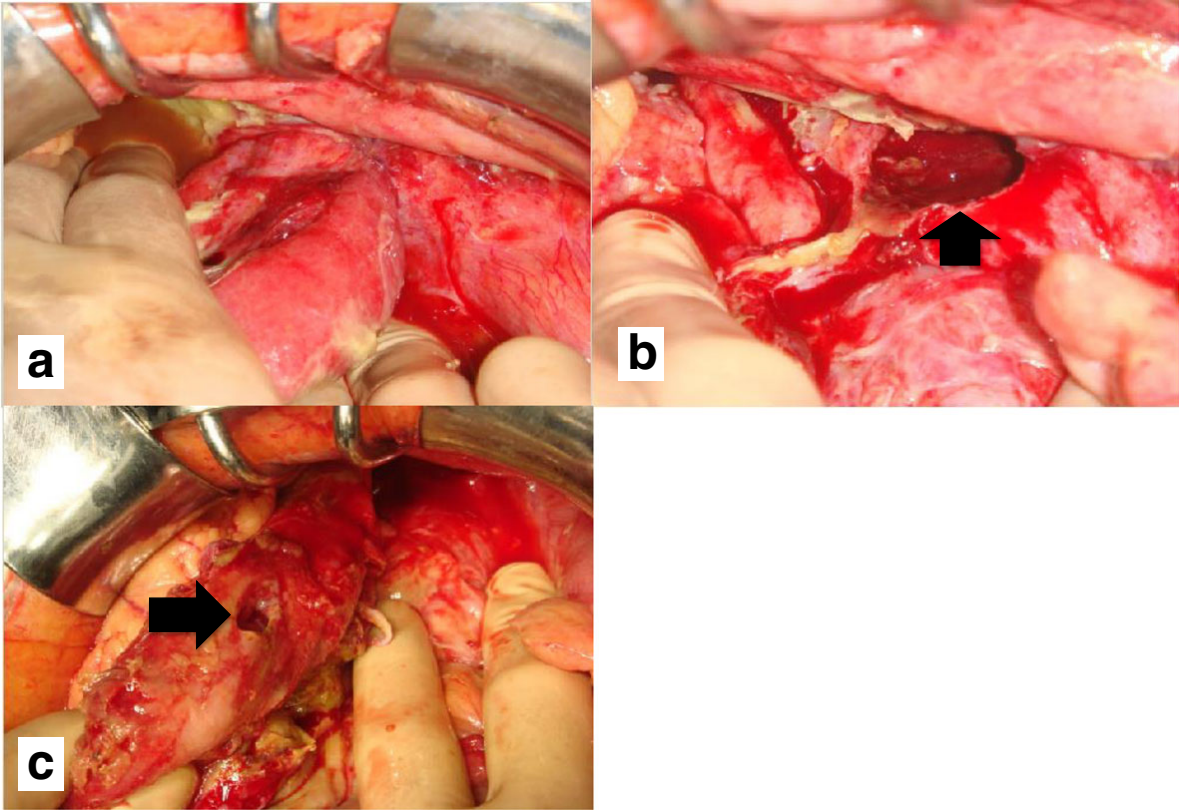
**a** It was revealed that the jejunum 100 cm from the Treitz ligament was herniated through the pelvic fracture with disruption of the posterior peritoneum. **b**: The hernia orifice was located at the site of disruption of the posterior peritoneum (*arrow*). **c**: The herniated jejunum exhibited ischemic changes and perforation (*arrow*)

A 65-year-old female was referred to a hospital because of the complaint of abdominal pain and was diagnosed with ileus. She developed ileus and high inflammatory response in serum laboratory data, so she was reduced the pressure in the gastric tube and started antibiotic treatment in another hospital. However, her general condition and ileus gradually deteriorated. Thereafter, she was presented at our department for further examination and treatment. She suffered from atypical psychosis, hyperthyroidism, and spinal canal stenosis. She had undergone four operations for spinal canal stenosis. On admission, her consciousness was disturbed and her blood pressure was low. Physical examination showed tenderness and muscular defense in her abdomen. The serum biochemical laboratory findings were as follows: white blood cells, 12,000/μl [normal range;4300-8000]; platelets, 34.5 × 10^3^/μl [18-34]; C-reactive protein (CRP), 28.05 mg/dl [0-0.4]; total bilirubin, 0.9 mg/dl [0.2-1.0]; aspartate aminotransferase (AST), 179 IU/l [13-33]; alanine aminotransferase (ALT), 197 IU/l [6-27]; γ-glutamyltransferase (γ-GTP), 211 IU/l [5-60]; creatinine kinase (CK), 204 IU/l [30-140]; lactate dehydrogenase (LDH), 436 IU/l [119-229]; and creatinine, 1.08 mg/dl [0.4-0.9]. Abdominal contrast-enhanced computerized tomography revealed left iliac fracture, extraintestinal free air, and herniation at the fracture site (Fig. 1). She was therefore diagnosed with bowel perforation due to a hernia at a pelvic fracture site. However, the time point of injury and fracture was not clear. We performed an emergency operation. It was revealed that the jejunum was herniated through the pelvic fracture with disruption of the posterior peritoneum, and that the herniated jejunum exhibited ischemic changes and was perforated (Fig. 2). During the operation, her vital signs were stable. We therefore did not perform damage control surgery and resected the segmental jejunum over a length of about 20 cm and constructed double-barreled jejunostomy. It was a state of septic shock and further contamination in the peritoneal cavity was high, so, we thought that a primary anastomosis was associated with high risk. The iliac bone fracture was fixed with four pins with percutaneous approach. Histopathologically, the herniated jejunum exhibited ischemic changes and perforation, and there was no malignancy in the specimen.

In the postoperative course, she was received antibiotics and vasopressors for septic shock. However, there was no need for a ventilator or dialysis or admission to ICU. In 7 days after operation, a residual abscess was revealed in the pouch of Douglas. We performed percutaneous drainage(Clavien-Dindo IIIa), and thereafter the patient’s general condition improved. Because the perforation was located 100 cm from the Treitz ligament, she did not get sufficient nutrition orally. She had to receive TPN. So, we performed jejunostomy closedown 35 days after the first operation. The postoperative course was without complication, but she was received rehabilitation until walking. She was discharged 64 days after the first operation (28 days after the second one).

## Discussion

The criteria of traumatic hernia proposed by Clain in 1964 are as follows: 1) the hernia must have appeared immediately after trauma and 2) the patient must have consulted a doctor soon enough for signs of the trauma to be identifiable [1]. However, many cases that did not fulfill these criteria have subsequently been reported. Therefore, Sahdev proposed new criteria of traumatic hernia in 1992 as follow: 1) the patient has no history of any hernia, 2) it is obvious that the patient has suffered an injury, 3) the appearance of herniation can occur at a delayed stage after trauma, and 4) a hernia sac can be present, as in this reported case [2]. The condition of the present case fulfilled these criteria proposed by Sahdev. In our case, although she had no history of high-energy injuries, she had hit her hip one month before presentation. Thus, we diagnosed traumatic hernia associated with pelvic fracture.

Traumatic hernia is rare, especially in association with pelvic fractures. To our knowledge, 19 cases have been reported (from a search of the PubMed database) (Table 1) [3–17]. In most reported cases, bowel entrapment or hernia was diagnosed either immediately or within a few days, but some cases were diagnosed at a delayed stage, namely, almost two to four weeks after injury. Adynamic ileus occurs in 5.5–18% of pelvic fractures [15]. The reason for it is retroperitoneal hematoma leading to intestinal dysfunction. It is difficult to distinguish herniation from adynamic ileus, therefore making a correct diagnosis is delayed in such cases. Seven of the cases shown in Table 1, resulted in a fatal outcome. Thus, surgeons treating patients with pelvic injuries should consider the possibility of any complications and perform a work-up examination in order to achieve an earlier accurate diagnosis. Of the 20 total cases (included our case), 13 were type A2 pelvic fracture under AO classification [18], 4 were type A1, 1 was type A3, and 1 was type C1. The possibility of traumatic hernia due to pelvic fracture needs to be considered as a possible complication of pelvic trauma. In particular, if the pelvic fracture type is A2, the bowels may herniate through the pelvic fracture with the disruption of the posterior peritoneum.

**Table 1:** Cases of bowel entrapment or herniation associated with pelvic fracture

Case	Author	Age/sex	Site of fracture	Pelvic fracture type^a^	Cause	Time to operation	Postoperative course	Outcome
	1 Arnold(‘07) [15]	76/F	ramus	A1	traffic accident	2 days	dead within hours	dead
	2 Derian(‘66) [3]	33/M	acetabulum	A2	traffic accident	immediately	uneventful	discharged after 6 weeks
	3 Lunt(‘70) [4]	17/F	pubic rami	A1	traffic accident	15 days	infection, ileus	discharged after 4 months
	4 Lunt(‘70) [4]	39/M	iliac wing	A1	traffic accident	10 days	leakage (re-operation)	discharged after 6 weeks
	5 Lunt(‘70) [4]	21/M	sacrum	A3	traffic accident	immediately	sepsis, renal dysfuction	dead after 11 weeks
	6 Lunt(‘70) [4]	17/M	acetabulum	A2	traffic accident	immediately	infection, hemiplegia	alive after 8 months
	7 Poilly(‘74) [5]	62/F	acetabulum	A2	traffic accident	12 days	dead on the next day	dead after 13 days
	8 Buchanan(‘80) [6]	13/M	acetabulum	A2	traffic accident	5 days	prolonged fever, hepatitis	discharged after 1 month
	9 Cotler(‘83) [7]	72/F	acetabulum, sacro-iliac joint	A2	traffic accident	4 days	sepsis, respiratory distress syndrome	dead after 1 week
	10 Lin(‘87) [8]	33/F	acetabulum	A2	traffic accident	4 weeks	sepitic shock, remove plate and screws	discharged after 12 months
	11 Ashai(‘88) [9]	29/M	acetabulum, ramus	A2	fall from a tree	3 days	sepsis, chest infection	discharged after 9 weeks
	12 Catsikis(‘89) [10]	36/F	sacro-iliac joint, iliac	C1	traffic accident	immediately	sepsis, renal dysfunction	dead after 7 days
	13 Kuhnke(‘89) [11]	20/F	sacrum	A3	traffic accident	4 weeks	sepsis, respiratory distress syndrome	discharged after 8 weeks
	14 Charnley(‘93) [12]	51/F	iliac wing	A2	traffic accident	3 weeks	uneventful	recovered
	15 Bacarese-Hamilton(‘91) [13]	80/F	acetabulum	A2	traffic accident	5 days	multi-organ failure	dead after 4 days
	16 Nasim(‘94) [14]	60/M	pubis rami	A1	fall	2 weeks	uneventful	unknown
	17 Stubbart(‘99) [15]	33/M	iliac wing	A2	traffic accident	4 weeks	leakage (re-operation)	discharged after 2 months
	18 Walcher(‘00) [16]	24/M	iliac wing	A2	traffic accident	5 days	uneventful	discharged after 5 weeks
	19 Kim(‘01) [17]	70/M	acetabulum, symphysis pubis	A2	fall from a tree	1 week	paralytic ileus	discharged after 6 months
	20 Our case	65/F	iliac wing	A2	unknown	1 month	residual abscess	discharged after 2 months

^a^AO classification

In our case, although the patient could not walk after her traumatic injury, her diagnosis was delayed due to a number of factors, including the patient’s introduction from another hospital, atypical psychosis, and her history of the spinal canal stenosis. If she had been promptly diagnosed with a pelvic fracture and had undergone pelvic fixation, it would have been possible to prevent the pelvic hernia at the fracture site. In our case, although her abdominal cavity was highly contaminated, we performed external fixation of the fractured iliac wing with bone pins at the same time. Because we thought the percutaneously inserted pins were not exposed the abdominal cavity directly.

However, we need to exercise caution with regard to pelvic fixation because cases have been reported in which the bowel was trapped at the fracture site, and in which the percutaneous iliosacral screws that were used for the fixation of a pelvic fracture were a possible reason for ileus, obstruction, and perforation [19].

## Conclusion

Traumatic hernia is rare, especially in association with pelvic fractures. Although its rarity, it follows a severe course. Thus, a proper diagnosis and effective treatment are necessary. Surgeons treating patients with pelvic injuries should consider the possibility of any complications and perform a work-up examination in order to achieve an accurate diagnosis at an earlier time point. If the pelvic bone is dislocated, the bowels may herniate through the pelvic fracture and disrupt the posterior peritoneum. We should therefore perform pelvic fixation in order to prevent the occurrence of pelvic hernia.

## Abbreviations

ALT: Alanine aminotransferase
AST: Aspartate aminotransferase
CK: Creatinine kinase
CRP: C-reactive protein
LDH: Lactate dehydrogenase
γ-GTP: γ-glutamyltransferase
